# Arthroscopic Repair of Posterior Root Tears of the Lateral Meniscus with All-Suture Anchor

**DOI:** 10.1016/j.eats.2021.12.037

**Published:** 2022-04-22

**Authors:** Filippo Familiari, Michelangelo Palco, Raffaella Russo, Gilbert Moatshe, Roberto Simonetta

**Affiliations:** a Department of Orthopaedics and Trauma Surgery, Magna Graecia University, Catanzaro, Italy; b Department of Biomedical, Dental and Morphological and Functional Images, Section of Orthopaedic and Traumatology, University of Messina, Messina, Italy; c Department of Orthopaedics and Trauma Surgery, Villa del Sole Clinic, Catanzaro, Italy; d Division of Orthopaedic Surgery, Oslo University Hospital, Oslo, Norway; e Oslo Sport Trauma Research Center, Norwegian School of Sports Science, Oslo, Norway

## Abstract

Meniscus root tears are increasingly being recognized and treated because of improved awareness and diagnostics. These injuries commonly occur in combination with knee ligament injuries. Untreated posterior meniscus root teats have been demonstrated to increase contact pressure and decrease contact area, ultimately leading to unfavorable joint loading and development of early osteoarthritis. Posterior lateral meniscus root tears (PLMRTs) also have been reported to increase anterior tibial translation and pivot shift in anterior cruciate ligament–deficient knees. Therefore, it is crucial to repair meniscal root tears when possible to restore knee joint loading and kinematics. Several techniques for repair of the PLMRT have been described. In this Technical Note, we describe our preferred technique for repair of PLMRT using an all-suture anchor. This technique is reproducible, does not need a tunnel, mitigates bungee effect of transtibial technique, and the anchor can easily be inserted on the footprint without a need for a guide.

Meniscus root tears are a specific type of meniscal injury that have been reported to account for 10% to 21% of all meniscal tears, affecting nearly 100,000 patients annually.^1^, ^2^, ^3^ Recently, Krych et al.^4^ reported on the incidence of posterior lateral meniscus root tears (PLMRTs) among 600 consecutive patients undergoing anterior cruciate ligament (ACL) reconstruction. The authors reported that 66% of the patients with acute ACL disruption had concomitant meniscal tears (31% had a lateral meniscal injury, 15% had a medial meniscal injury, and 20% had both medial and lateral meniscal injuries). The most common lateral meniscal tear was a posterior horn lateral meniscal oblique radial tear (LMORT), which accounted for 18% of all meniscal tears in ACL-injured knees. Overall, the incidence of ACL injury with a concomitant posterior horn LMORT was 12%.

In 2011, Forkel and Petersen^5^ described 3 types of lesions of the posterior horn of the lateral meniscus: (1) the avulsion of the root at the plateau of the tibia with an intact meniscofemoral ligament, (2) a radial tear of the posterior horn of the lateral meniscus with an intact meniscofemoral ligament, and (3) a complete injury of the posterior horn of the lateral meniscus with rupture of the meniscofemoral ligament. Meniscal root tears have been further classified into 5 types: (1) partial root tear; (2) complete radial root tear which is further subdivided based on its location from the root attachment (0 mm to less than 3 mm [subtype 2A], 3 mm to less than 6 mm [subtype 2B], and 6 to 9 mm [subtype 2C]); (3) complete root tear with a bucket handle meniscus tear; (4) oblique tear into the root attachment; and (5) root avulsion fracture.^6^ Krych et al.^4^ described 4 types of posterior horn LMORTs including a corresponding treatment for each. The tear types were (1) partial oblique tear <10 mm from the lateral root (type 1); (2) complete oblique tear <10 mm from the root attachment, but not involving the root site (type 2); (3) incomplete oblique tear extending >10 mm from the root (type 3); and (4) complete oblique tear extending >10 mm from the root (type 4). Type 1 tears were repaired with partial meniscectomy. Type 2 tears were repaired with transtibial pull-out repair. Type 3 and type 4 tears were repaired with all-inside suture repair or, rarely, with inside-out suture repair.

Untreated meniscal root tears have been reported to result in altered joint biomechanics and accelerated articular cartilage degeneration. In this regard, meniscal root tears increase articular cartilage contact pressure and accelerate degenerative changes.^7^, ^8^, ^9^, ^10^, ^11^ Furthermore, LMPRTs have been shown to increase anterior tibial translation and pivot shift in ACL-injured knees.^12^ Therefore, untreated PMLRTs can lead to persistent instability and increased forces on the reconstructed ligaments, ultimately leading to failure. Several repair techniques for PLMRT have been described, including transtibial suture technique, suture anchor technique, and the all-inside technique for Forkel type 2 lesions. The different techniques have different advantage and disadvantage profiles, and it is important for surgeons to be familiar with different techniques of addressing these often-challenging injuries. This Technical Note describes our surgical technique to repair PLMRTs using an all-suture anchor (Video 1).

## Surgical Technique (With Video Illustration)

### Indications and Contraindications

Repair of the PLMRT is indicated in patients with or without concomitant cruciate ligament tears including ACL, posterior cruciate ligament, and in multiligament injuries. Contraindication for the repair of PLMRT is advanced knee osteoarthritis (Outerbridge grade 3 and 4). When considering age, joint age (biological age), concomitant injuries, and general patient activity and function level are more important than chronological age. The role of alignment and alignment correction with concurrent meniscal root repair has not been elucidated.

### Patient Positioning and Anesthesia

**Fig 1:**
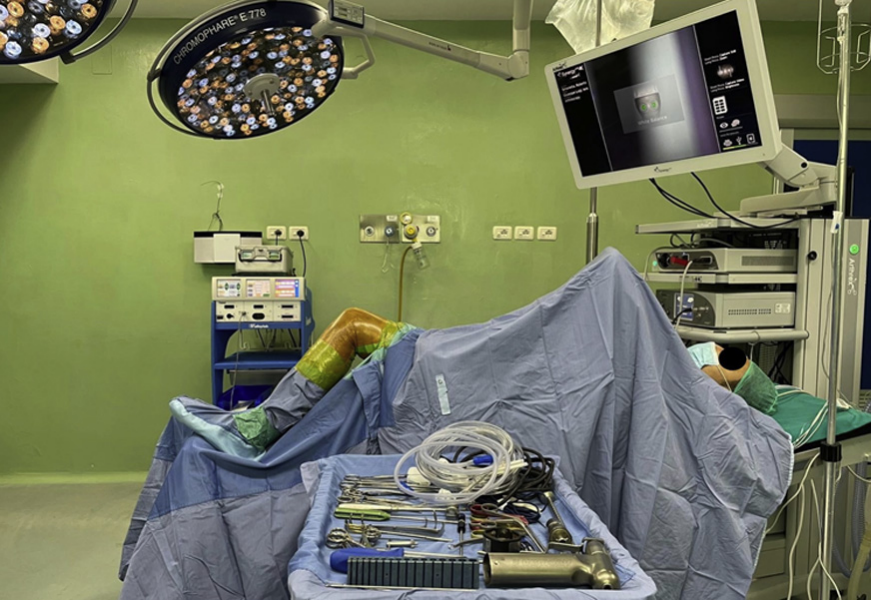
Patient positioning and room setup for knee arthroscopy.

The surgical procedure is performed with the patient under epidural anesthesia in the supine position. A bilateral knee examination is performed to evaluate for any concurrent ligamentous instability and to assess range of motion. A padded, nonsterile tourniquet is placed high on the operative thigh, and the patient is positioned in the standard arthroscopy position, with lateral support at the level of the tourniquet and a foot post to allow the knee to be maintained at 90° of flexion when required. The operative leg is prepared and draped in standard fashion (Fig 1).

### Tear Assessment and Portal Placement

**Fig 2:**
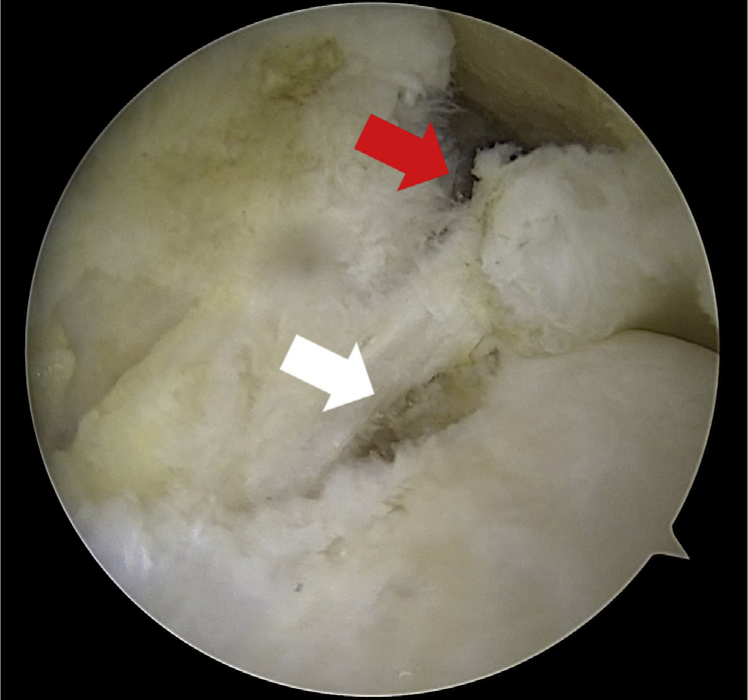
Intra-articular view of the left knee through the standard anterolateral portal showing the torn lateral meniscus root (red arrow) and its root attachment (white arrow) after debridement performed with a burr.

**Fig 3:**
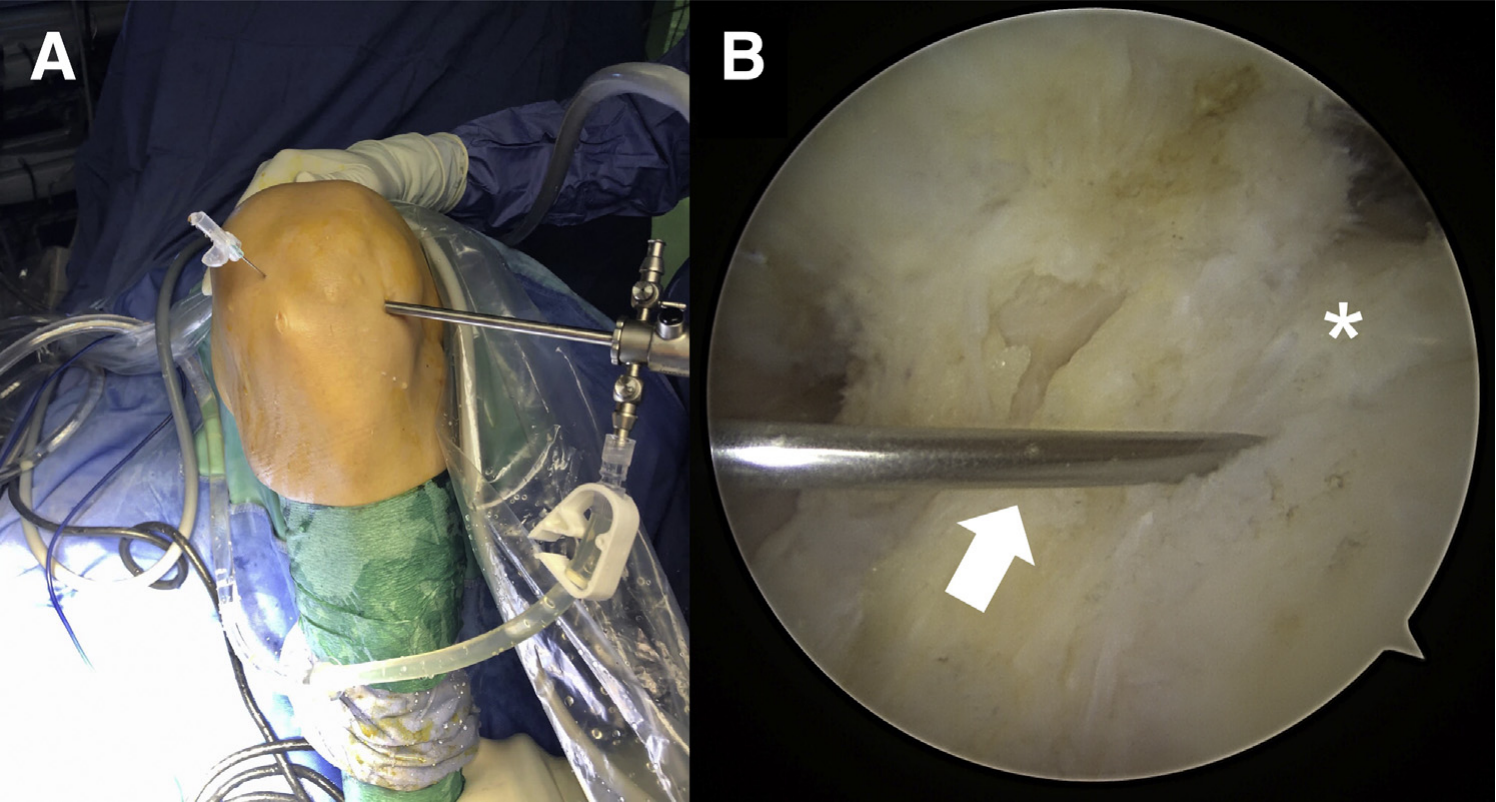
(A) Extra-articular view of the left knee. With the knee hyperflexed, a needle is placed higher than the standard anteromedial portal to establish the correct position of the accessory portal. (B) Intra-articular view of the left knee through the standard anterolateral portal. The lateral meniscus root attachment (asterisk) and the tip of the needle (white arrow) are shown under arthroscopic visualization.

Standard anterolateral and anteromedial portals are created for routine diagnostic arthroscopy. The torn meniscal root should be probed to assess for severity and tear pattern.^6^ PLMRTs can be easily identified in case of a concomitant ACL injury. A grasper is used to assess position the torn meniscal root, meniscus mobility, and determine the ideal location to perform the repair. With a curved curette or a burr, the tibial attachment site of the torn meniscus root is debrided of soft tissues and overlying cartilage down to a bleeding bone bed to improve healing of the repair (Fig 2). Furthermore, appropriate knee flexion is key to achieve a correct anchor placement. Therefore, an accessory high anteromedial portal is created with the knee hyperflexed (Fig 3). If the lesion is classifiable as a type 2 root lesion according to Forkel and Petersen,^5^ we prefer use a side-to-side suture; if not, we use FiberTak Soft Anchor (Arthrex, Naples, FL).

### Suture Anchor Placement

**Fig 4:**
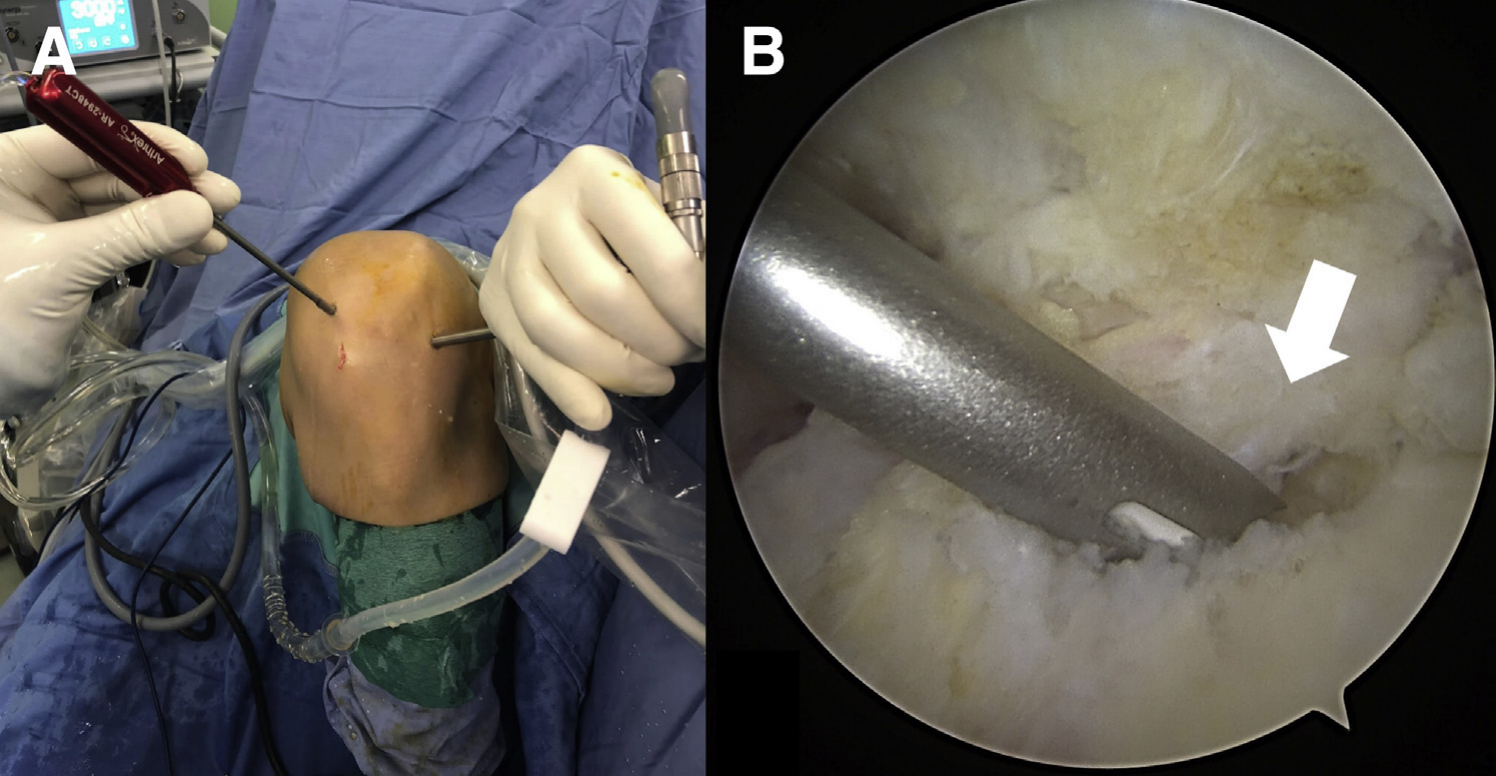
(A) Extra-articular view of the left knee. With the knee hyperflexed, the curved guide is introduced through the accessory portal. (B) Intra-articular view of the left knee through the standard anterolateral portal. The tip of the curved guide is placed at the prepared insertion site of the footprint (white arrow).

**Fig 5:**
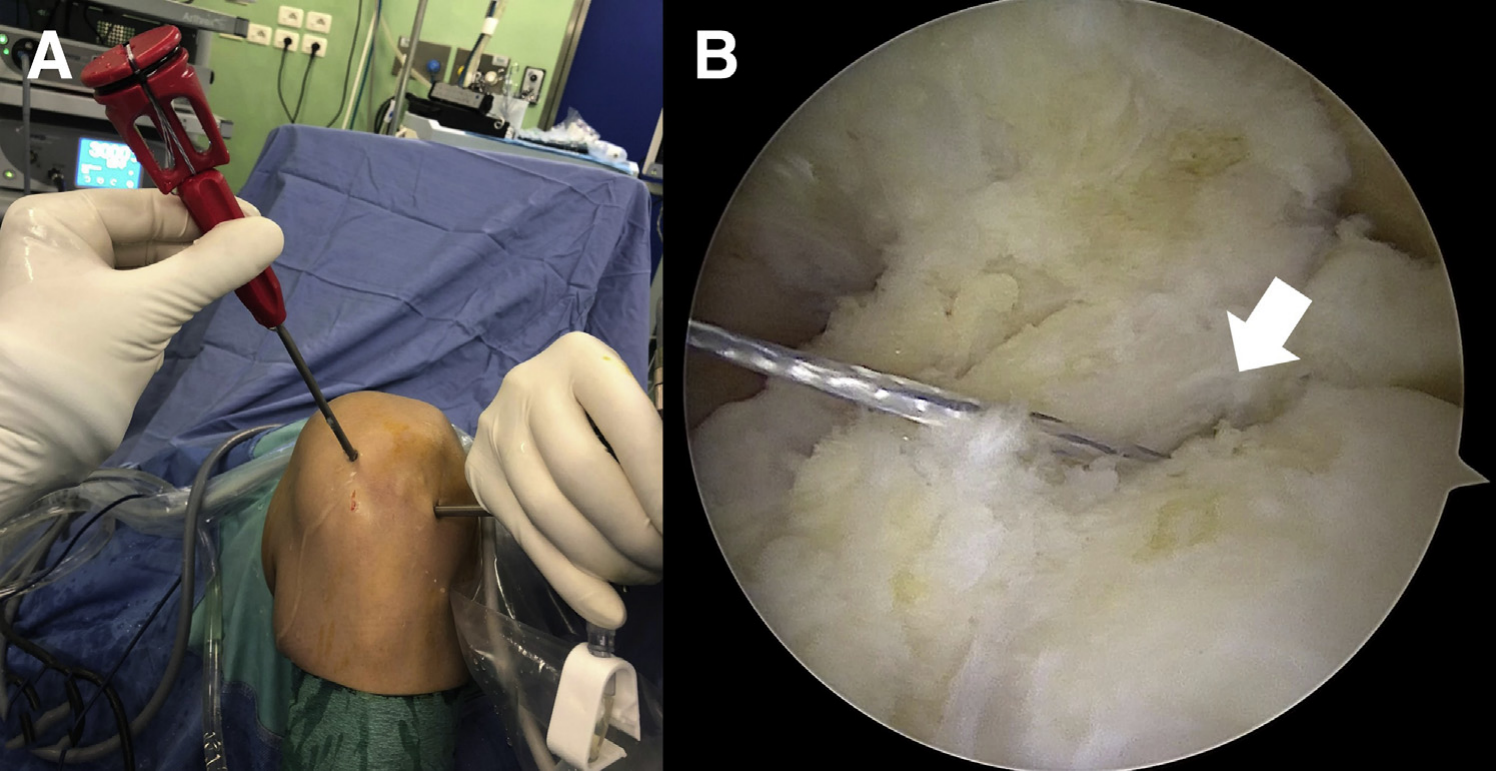
(A) Extra-articular view of the left knee. With the knee hyperflexed, the 1.8-mm FiberTak Soft Anchor is placed through its curved guide. (B) Intra-articular view of the left knee through the standard anterolateral portal showing the 1.8-mm FiberTak Soft Anchor loaded with a FiberWire no. 2 (white arrow).

With the knee at 90° of flexion, a curved guide (AR-2948 CT; Arthrex) is inserted through the high anteromedial portal and the guide is placed at the prepared insertion site of the footprint (Fig 4). A pilot hole is created at an appropriate angle using a 1.8-mm flexible drill (AR-3600ND-2; Arthrex). Afterwards, a 1.8-mm FiberTak Soft Anchor (AR-3600; Arthrex) loaded with a FiberWire no. 2 is placed (Fig 5). The authors recommend to set the anchor and test its stability by pulling on the sutures.

### PLMRT Suture

**Fig 6:**
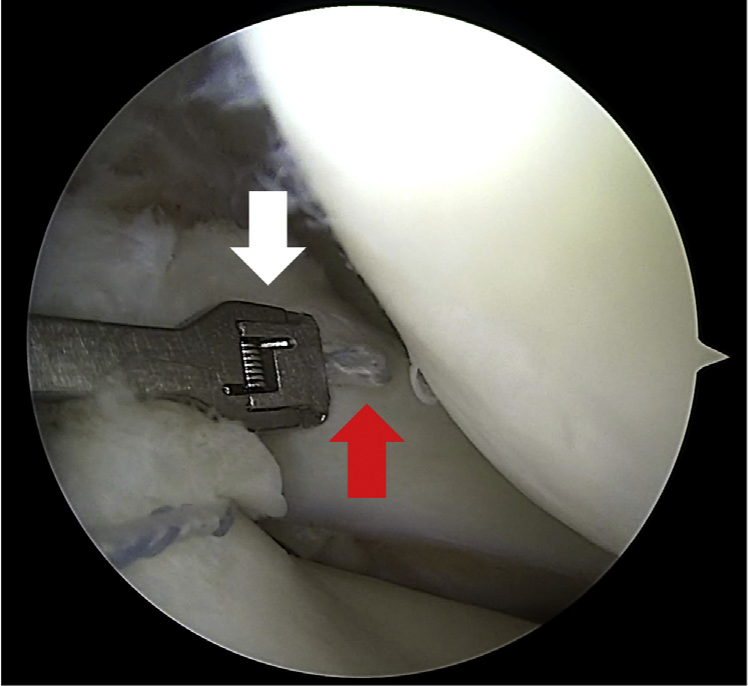
Arthroscopic view of the left knee through the standard anterolateral portal with knee in figure-of-4 position showing the suture (red arrow) captured in the top jaw (white arrow) of the Knee Scorpion introduced through the standard anteromedial portal.

**Fig 7:**
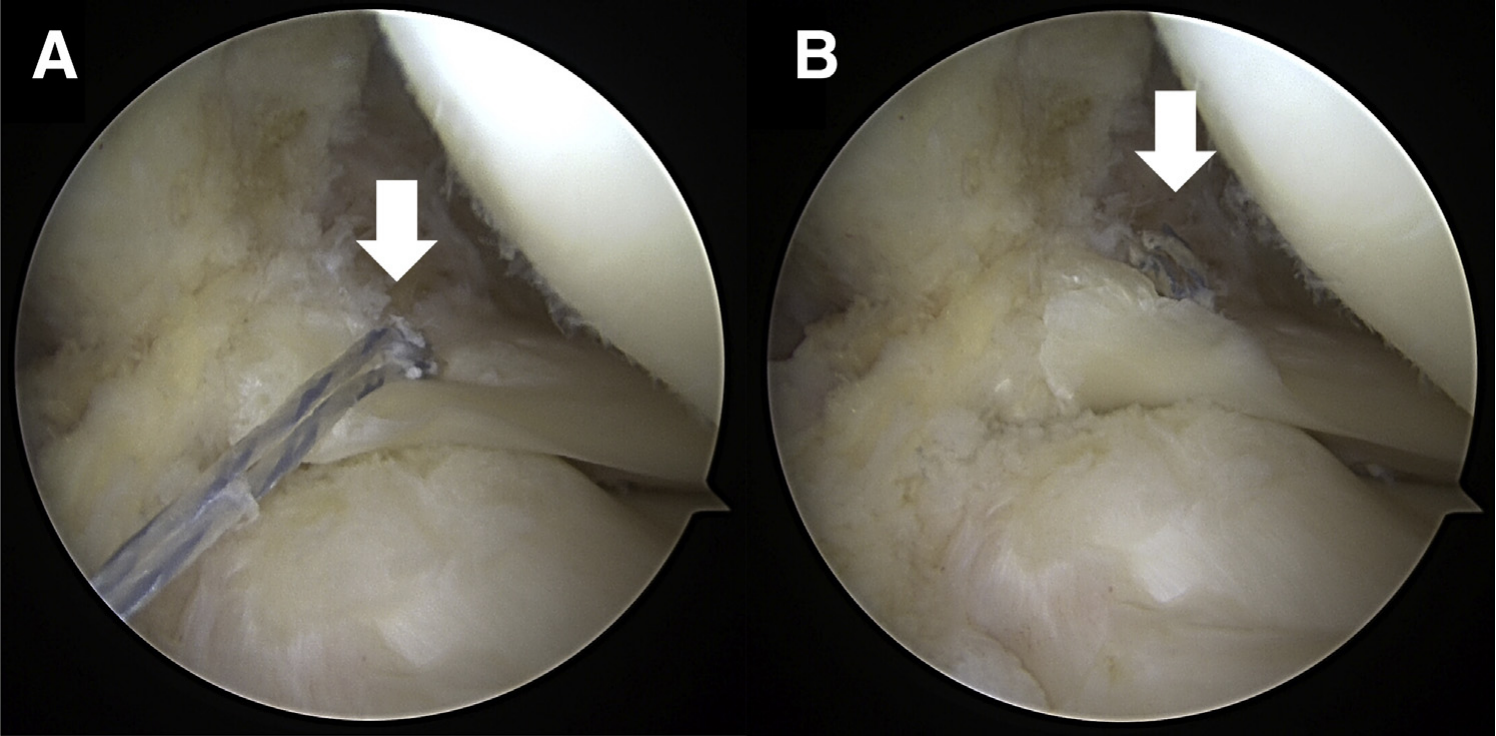
Arthroscopic view of the left knee through the standard anterolateral portal with knee in figure-of-4 position showing the suture (white arrow) tightened (A) and then cut (B) with a FiberTape Cutter through the standard anteromedial portal.

The leg is brought into the figure-of-4 position applying a varus stress to open up the lateral compartment. The sutures are retrieved through the standard anteromedial portal with a Suture Retriever (Arthrex). A direct suture passer and retriever (Knee Scorpion; Arthrex) is loaded into the bottom jaw, and it is then introduced through the standard anteromedial portal. Once the suture passer and retriever (Knee Scorpion; Arthrex) has been positioned at the desired location on the meniscus, the top jaw is closed and the back trigger is squeezed to advance suture through the tissue bottom up. With the suture captured in the top jaw (Fig 6), the Knee Scorpion is retracted from the tissue. The same procedure is repeated with the second, free-end of the suture. A simple arthroscopic knot is then tightened under direct arthroscopic visualization (Fig 7). Finally, with a FiberTape Cutter (Arthrex), the FiberWire is cut and the strength of the suture fixation is tested with a probe. Pearls and pitfalls are summarized in Table 1.

**Table 1:** Pearls and Pitfalls

Pearls	Pitfalls
High accessory medial portal to place the suture anchor	Low portals can make it difficult to visualize the footprint and place the anchor
Ensure the meniscus end at the tear can be mobilized to the footprint	The anchor is laced in the nonanatomic position
Remove the soft tissue and cartilage at the insertion site to enhance healing	The soft tissue and cartilage at the insertion site are not removed, impairing healing
Repair the meniscal root before fixing the ACL graft to have adequate room in cases of concurrent ACL reconstruction	Fixing the ACL before the meniscal root repair will make the working space in the notch smaller, and varus forces during repair may be detrimental to the ACL graft
Tension the suture under direct arthroscopic visualization	Not having enough tension on the suture may impair the stability and healing of the repair
Protect the repair by delaying weight-bearing	Early weight-bearing can be detrimental to the repair because of increased hoop stresses

ACL, anterior cruciate ligament.

### Rehabilitation Protocol

Physical therapy should start as soon as possible after surgery, which should include early passive range of motion exercises in a safe zone of 0° to 90°of flexion for the initial 2 weeks. After 2 weeks, patients can work on further increases in knee flexion as tolerated. In the condition of non–weight-bearing, a knee brace is not necessary. Patients should remain non–weight-bearing for 4 weeks. Progressive advancement to full weight-bearing begins at 4 weeks. Recovery of muscular endurance, strength, and power is encouraged, especially active quadriceps exercises. Deep leg presses and squats greater than 70° of knee flexion should be avoided for at least 4 months after surgery.

## Discussion

The purpose of this Technical Note was to describe our preferred surgical technique that we use routinely to repair PLMRTs. The most-often used techniques for repair of these lesions are the transtibial pull-out technique suture and the side-to-side suture.^13^, ^14^, ^15^, ^16^, ^17^, ^18^, ^19^ Padalecki et al.^20^ and LaPrade et al.^21^ underlined the importance to restore the native anatomy of root attachment on human cadaveric knees. The transtibial pull-out technique involves passing sutures through posterior lateral meniscal root, getting back them through transtibial tunnels drilled from the anteromedial surface of the proximal tibia to the posterior meniscal root footprint, and then tying them over a button.^22^, ^23^, ^24^, ^25^, ^26^ The main advantage of using a soft anchor to repair PLMRTs is no need of tibial tunnels, making it a less-invasive procedure and no risk of tunnels collision in case of concurrent ligament surgery (e.g., ACL reconstruction) or corrective osteotomies. This is particularly important when considering that 8% to 10% of cases with an ACL tear have a concomitant PLMRT^13^^,^^27^^,^^28^; and it is crucial to avoid collision of tunnels in case it is necessary to treat both lesions.^29^ In addition, it is simpler to identify posterolateral root attachment and at the same time place the anchor, rather than use an external guide or a pin guide to aid pin placement. The compartment may be narrow, making it difficult to place the pin guide, and the pin does not always exit at the desired position. Another advantage of this technique is that it is easier to tension under direct arthroscopic visualization and there are no knots in the joint. Finally, FiberTak Soft Anchor has no need of hardware like button or screw for tibial fixation. And besides, the stability and the strength of fixation are not influenced by the use of the FiberTak Soft Anchor.^30^ In literature it has been reported^31^ that a similar soft anchor had pull-out force of 290.5 (± 15.3 DS N), which is greater than the mean pull-out failure strength of posterior horn of meniscus (71.6 ± 23.2 DS N).^32^ Advantages and disadvantages are summarized in Table 2.

**Table 2:** Advantages and Disadvantages

Advantages	Disadvantages
No need for hardware	Cost
No risk of tunnel collision	Hard to work with an intact ACL
Tensioning on direct arthroscopic visualization	
Easy placement of anchor at the footprint	
Strong fixation	

ACL, anterior cruciate ligament.

Ohori et al.^33^ compared the effect of the lateral meniscus complete radial tear at different tear sites on the load distribution and transmission functions. The authors demonstrated that the detrimental effect (i.e., greatest peak contact pressure and lowest contact area) was greater as the tear site was closer to the lateral meniscus posterior root in the deep-flexed position. With the aim to restore knee kinematic, various repair techniques of the LMPR have been described, including root fixation performed through side-to-side repair and transosseous tunnels.^13^, ^14^, ^15^, ^16^, ^17^, ^18^, ^19^^,^^34^ A technique using suture anchors has also been described by several authors.^34^, ^35^, ^36^ It has been demonstrated that the different repair techniques are significantly weaker than native LMPR in terms of elongation, dynamic stiffness, and load-to-failure.^30^ The anchor fixation technique showed significantly less elongation (0.8 mm −0.22/+1.08 mm) compared with the transtibial pull-out fixation (1.3 mm −0.98/+2.06 mm); about dynamic stiffness, no statistical differences were observed between suture anchor repair and transtibial repair of LMPR tears. Finally, at load-to-failure test, the suture anchor fixation revealed stronger than transtibial technique (120 N vs 50 N), but weaker than native posterior root (120 N vs 360 N). Therefore, rehabilitation protocols should aim at protecting the repair against elongation and pull-out (failure) while the repair is healing.

This technique cannot be used to type 2 root lesions (according to classification by Forkel and Petersen^5^), in which case a side-to-side suture is a better option. In our opinion, the only case in which is possible to make a side-to-side suture is when there is a type 2 root lesion, and in addition when the fragment attached to the root is large enough to be purchased by the needle and suture. In this case, side-to-side repair is theoretically biomechanically superior due to the maintenance of meniscal native physiologic properties.^13^^,^^15^^,^^17^, ^18^, ^19^ This procedure for PLMRTs repair is reproducible and easy to perform for both experienced and less-experienced surgeons.

Untreated LMPRTs increase the risk of graft failure in ACL-reconstructed knees and increased risk of developing early osteoarthritis due to increased contact pressure in the lateral compartment. Treating meniscal root tears has been demonstrated to restore knee kinematics in biomechanical studies and delay the progression of osteoarthritis in clinical studies. Despite several techniques being described, none has been shown to be superior. It is important to fix the meniscal root to the native meniscal root footprint to restore both the kinematics and contact pressures.^37^, ^38^, ^39^ Nonanatomic repair has been reported to not restore joint contact pressures. Furthermore, it is important to enhance tissue healing and protect the repair while healing.

## Conclusions

In conclusion, we encourage to use routinely the aforementioned surgical technique for treatment of PLMRTs, because of it is minimally invasive, simple, effective, and reproducible.

## Supplementary Data

ICMJE author disclosure forms

Video 1 We present our arthroscopic repair of posterior root tears of the lateral meniscus with all-suture anchor. The surgical procedure is performed with the patient under epidural anesthesia in the supine position. A padded, nonsterile tourniquet is placed high on the operative thigh, and the patient is positioned with lateral support at the level of the tourniquet and a foot post to allow the knee to be maintained at 90° of flexion when required. The operative leg is prepared and draped in standard fashion. Standard anterolateral and anteromedial portals are created for routine diagnostic arthroscopy. The torn meniscal root should be probed to assess for severity and tear pattern. First with a burr and then with a curved curette, the tibial attachment site of the torn lateral meniscus root is debrided of soft tissues and overlying cartilage down to a bleeding bone bed to improve healing of the repair. Appropriate knee flexion is key to achieve a correct anchor placement. Therefore, an accessory high anteromedial portal is created with the knee hyperflexed. A curved guide is inserted through the high anteromedial portal and the guide is placed at the prepared insertion site of the footprint. A pilot hole is created at an appropriate angle using a 1.8-mm flexible drill. Afterwards, a 1.8 mm all-suture anchor loaded with a FiberWire no. 2 is placed. We recommend to set the anchor and test its stability by pulling on the sutures. The leg is then brought into the figure-of-4 position. The sutures are retrieved through the standard anteromedial portal. A direct suture passer and retriever is loaded into the bottom jaw, and it is then introduced through the standard anteromedial portal. Once the suture passer and retriever has been positioned at the desired location on the meniscus, the top jaw is closed and the back trigger is squeezed to advance suture through the tissue bottom up. With the suture captured in the top jaw, the Knee Scorpion is retracted from the tissue. The same procedure is then repeated in the posterior part of the meniscus with the second, free-end of the suture. A simple arthroscopic knot is then tightened under direct arthroscopic visualization aiming for the correct amount of tension. Finally, with a FiberTape Cutter, the FiberWire is cut and the strength of the suture fixation is tested with a probe.
